# Nitric oxide and pulmonary arterial hypertension

**DOI:** 10.21542/gcsp.2017.14

**Published:** 2017-06-30

**Authors:** Adrian H. Chester, Magdi H. Yacoub, Salvador Moncada

**Affiliations:** 1 National Heart & Lung Institute, Imperial College London, Heart Science Centre, Harefield, Middlesex, UB9 6JH, United Kingdom; 2 School of Medical Sciences, Manchester Cancer Research Centre, University of Manchester, Wilmslow Road, Manchester, M20 4QL, United Kingdom

## Abstract

The pathogenesis of pulmonary arterial hypertension remains undefined. Changes in the expression and effects mediated by a number of vasoactive factors have been implicated to play a role in the onset and progression of the disease. The source of many of these mediators, such as nitric oxide (NO), prostacyclin and endothelin-1 (ET-1), is the pulmonary endothelium. This article focus in the role of nitric oxide in PAH, reviewing the evidence for its involvement in regulation of pulmonary a vascular tone under physiological conditions, the mechanisms by which it can contribute to the pathological changes seen in PAH and strategies for the use of NO as a therapy for treatment of the disease.

## Pulmonary arterial hypertension

**Figure 1.**
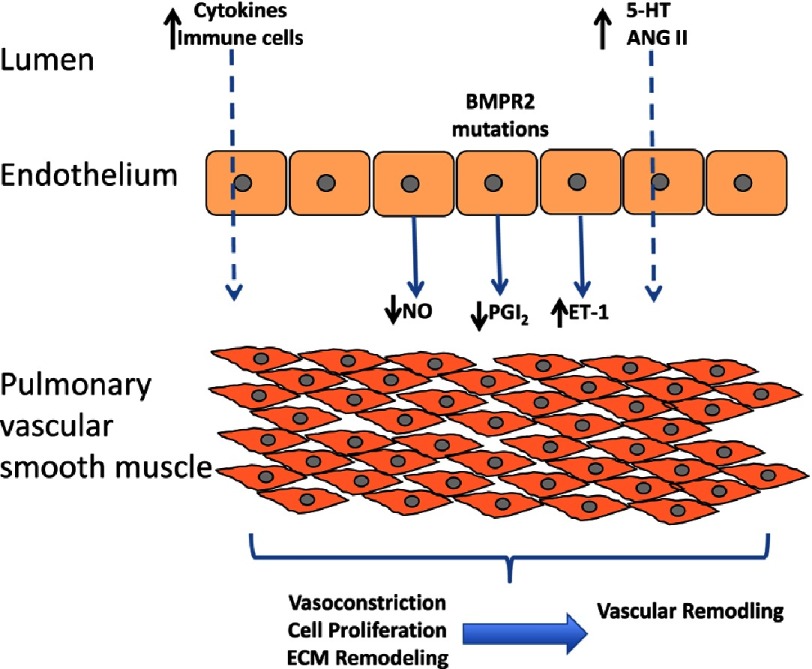
Schematic diagram of the release of vasoactive factors from the endothelium and their action on the underlying vascular smooth muscle.

Pulmonary arterial hypertension (PAH) is a rare, debilitating condition with a poor prognosis. While the precise mechanism(s) that mediated the onset and progression of the disease remain undefined, several factors have been implicated in the pathology of PAH. These include endothelial dysfunction, oxidant stress, metabolic dysfunction, immune dysregulation and genetic factors^1–9^, all of which can contribute to the pulmonary artery vasoconstriction, vascular remodelling and right ventricular failure that are features of the disease (Figure 1).

### Epidemiology of PAH

PAH has an incidence of 15–50 people per million. Initially, median survival was calculated to be only 2.8 years^10,11^. More recently, data has shown that depending on the presence of co-morbidities the survival 3 years after diagnosis is between 54.4% and 58.2%^12^. One year survival of PAH has been shown to be influenced by a range of prognostic indicators including renal insufficiency, PAH associated with connective tissue disease, functional class III heart failure, mean right atrial pressure, resting systolic blood pressure, heart rate, 6-minute walk distance, brain natriuretic peptide levels, percentage predicted carbon monoxide diffusion capacity and pericardial effusion on echocardiogram^13^. There is a predominance of the condition in women, which varies according to the aetiology of the disease^14^.

### Pathogenesis of PAH

The aetiology of PAH is varied, this is reflected in the World Health Organisation’s clinical classification of pulmonary hypertension (Table 1)^15^. Despite the wide range of causative factors, the lungs of patients with pulmonary hypertension exhibit a range of classical histological changes. These include remodelling of the pulmonary vessels, regions of neovascularisation, fibrotic changes in the vessel wall, thrombus formation and formation of plexiform lesions^16^. Plexiform lesions are composed of proliferating endothelial cells, matrix proteins and fibroblasts that obliterate the vascular lumen^17^. The reasons for their formation are poorly understood, however hypoxia, inflammation, shear stress, drugs, viral infections and genetic susceptibility have all been implicated^18^.

**Table 1:** WHO classification of pulmonary hypertension.

Group 1	Pulmonary arterial hypertension (PAH)
	Idiopathic (IPAH)
	Heritable (HPAH)
	Bone morphogenetic protein receptor type 2 (BMPR2)
	Activin receptor-like kinase 1 gene (ALK1), endoglin (with or without haemorrhagic telangiectasia)
	Unknown
	Drug- and toxin-induced
	Associated with (APAH):
	Connective tissue diseases
	Human immunodeficiency virus (HIV) infection
	Portal hypertension
	Congenital heart disease (CHD)
	Schistosomiasis
	Chronic haemolytic anaemia
	Persistent pulmonary hypertension of the newborn (PPHN)
Group 1′	Pulmonary veno-occlusive disease (PVOD) and/or pulmonary capillary haemangiomatosis (PCH)
Group 2	Pulmonary hypertension due to left heart diseases
	Systolic dysfunction
	Diastolic dysfunction
	Valvular disease
Group 3	Pulmonary hypertension due to lung diseases and/or hypoxemia
	Chronic obstructive pulmonary disease (COPD)
	Interstitial lung disease (ILD)
	Other pulmonary diseases with mixed restrictive and obstructive pattern
	Sleep-disordered breathing
	Alveolar hypoventilation disorders
	Chronic exposure to high altitude
	Developmental abnormalities
Group 4	Chronic thromboembolic pulmonary hypertension (CTEPH)
Group 5	PH with unclear multifactorial mechanisms
	Haematological disorders: myeloproliferative disorders, splenectomy
	Systemic disorders: sarcoidosis, pulmonary Langerhans cell histiocytosis, lymphangioleiomyomatosis, neurofibromatosis, vasculitis
	Metabolic disorders: glycogen storage disease, Gaucher disease, thyroid disorders
	Others: tumoral obstruction, fibrosing mediastinitis, chronic renal failure on dialysis

A number of factors and agents responsible for initiating and progressing the increases in pulmonary artery pressure have been suggested. Given the variety of different forms of the disease, it’s not surprising that so many different mediators and mechanisms are believed to be responsible (Table 2), many of which have been reviewed elsewhere^1–5,19^. At the cellular level dysfunction of the pulmonary endothelium seems to underpin many of the changes seen in PAH. Endothelial cells regulate vascular tone, vascular remodelling and inflammation via the release a range of vasoactive molecules that interact with blood elements and the underlying vascular smooth muscle. These mediators include nitric oxide (NO), prostacyclin and endothelin-1 (ET-1). The role of both ET-1 and prostacyclin has recently been reviewed in this journal^2,3^. The focus of the present article is on the role of NO in the onset and progression of PAH as well as the use of NO therapies for the alleviation of the clinical symptoms and improving the quality of life of patients with PAH.

**Table 2:** Causative agents associated with the pathogenesis of PAH.

Chemical / Drug mediators	Associated conditions
Aminorex,	Mutations in bonemorphogenic protein receptor 2
Fenfluramine,	Systemic sclerosis
Dexfenfluramine,	HIV infection
Cocaine,	Portal hypertension
Phenylpropanolamine	Congenital heart disease with left-to-right shunts
St. John’s Wort	Recent acute pulmonary embolism
Chemotherapeutic agents	Sickle cell disease
Serotonin re-uptake inhibitors	
Amphetamines	
Metamphetamines and L-tryptophan	
Exposure to chemicals such as toxic rapeseed oil	

## Nitric oxide in the physiology of the pulmonary circulation

As with all other vascular beds, the production of NO by the pulmonary endothelium helps to regulate vascular tone. While a diverse range of endogenous chemical mediators have been identified to stimulate the release of NO from endothelial cells, the frictional force of the flow of blood over the surface of the endothelial cells (shear stress) is the principal physiological stimulus for NO release^20^.

**Figure 2.**
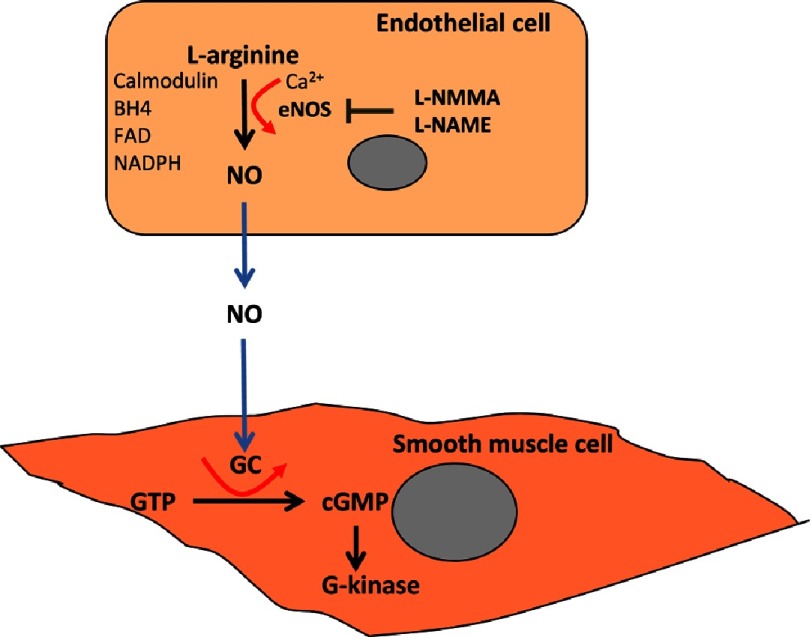
Diagram showing the mechaism of synthesis of nitric oxide from endothelail cells and its action on vascular smooth muscle cells.

The role of endothelial cells in regulating vascular tone was first reported as the “obligatory role” of the vascular endothelium in mediating relaxation of the underlying smooth muscle in 1980 with the discovery of endothelium-derived relaxing factor (EDRF)^21^. It was not until 7 years later that the chemical nature of EDRF as NO was identified and the metabolic pathway leading to its synthesis elucidated. Nitric oxide is formed by the enzymatic cleavage of the terminal amino group from the amino acid L-arginine^22,23^. The enzyme, termed nitric oxide synthase (NOS) was specific for the L isomer, but had no activity against the D isomer of arginine (Figure 2). At approximately the same time, NO was also shown to account for the biological actions of nitrovasodilators, which are capable of generating NO from their chemical structure (so called NO donors)^24^.

The characterisation of the first of a number of inhibitors of NOS, such as L-N^G^-monomethyl Arginine (L-NMMA), L-N^G^-Nitroarginine methyl ester (L-NAME) and N^G^-Methyl-L-arginine (L-NMA), allowed researchers to probe the role of NO in the vascular system, leading to the first demonstration that inhibition of NO increases blood pressure in animals and induces vasoconstriction in human blood vessels^25–27^. In addition, it was shown that the effects of NO are not confined to the vascular system alone. Besides the endothelial NOS two additional isoforms of the NOS enzyme, a neuronal isoform and an inducible isoform, have been identified. These three isoforms release NO for a variety of different biological functions^28^. All NOS enzymes bind calmodulin and contain haem and utilise L-arginine and molecular oxygen as substrates and requires the cofactors reduced nicotinamide-adenine-dinucleotide phosphate (NADPH), flavin adenine dinucleotide (FAD), flavin mononucleotide (FMN), and (6R-) 5,6,7,8-tetrahydrobiopterin (BH4). They differ in their distribution, dependence on calcium and the amount of NO that they produce (Table 3)^29^.

**Table 3:** Characteristics NOS enzymes.

Name	Gene	Location	Co-factors
NOS1, nNOS, Neuronal NOS	Chromosome 12	Neuronal tissue, skeletal muscle	Heam, BH_4_, NADPH, Ca^2 +^
NOS2, iNOS, Inducible NOS	Chromosome 17	Immune system Cardiovascular system	Heam, BH4, NADPH,
NOS3, eNOS, Endotheial NOS	Chromosome 7	Endothelium	Heam, BH_4_, NADPH, Ca^2 +^

Most of the biological actions of NO depend on its ability to stimulate the enzyme soluble guanylate cyclase with the subsequent formation of cyclic 3′, 5′ guanosine monophosphate (cGMP)^30^. This leads to the activation of cGMP-dependent kinases (cGKs), which leads to the activation myosin phosphate and a subsequent release of calcium from intracellular stores allowing smooth muscle cells to relax. cGKs may also activate transcription factors that may change gene expression in the cell and mediate other responses of the cell to NO or alter its response to other stimuli^31,32^. The bioavailability of NO is influenced by its inactivation by excessive amounts of oxygen-derived species such as superoxide (O_2_^−^) or changes in the protective effects of endogenous antioxidants.

Sources of O_2_^−^ include NADPH oxidases, xanthine oxidase, cytochrome P450 enzymes and from complexes I and III of the electron transport chain in mitochondria. An additional source of O_2_^−^ may come from uncoupling of NOS, which has been suggested to occur in several pathological conditions^33–35^. Unless inactivated by oxygen free radicals, the effects of NO are terminated by the breakdown cGMP by phosphodiesterase (PDE) enzymes. There are a number of different isoforms of PDEs that have specific distributions in different organs and vascular beds^36^.

### Interactions between nitric oxide and other endothelium-derived vasoactive mediators

The endothelium was initially regarded simply as monolayer of cells that line the vasculature serving as a barrier between the blood and the vessel wall^37^, however it is now considered more as an organ in its own right that regulates vascular homeostasis. In addition to NO, the endothelium also releases prostacyclin (PGI_2_) and ET-1, which also both have a direct effect on the vessel wall^38–41^. ET-1 is a powerful vasoconstrictor peptide that can be considered as a functional antagonist to the vasodilator effects of NO. ET-1 has also been implicated in the pathogenesis of PAH and inhibition of its effects is a pharmacological target for the treatment of the disease^42,43^. In contrast, PGI2 is a vasodilator and anti-aggregating substance. The effect of prostacyclin on platelet aggregation are enhanced by the presence of NO^44^. The effects of PGI2 are mediated via its action on cell surface receptors that in turn stimulate the accumulation of cAMP. Administration of PGI2, has been the most widely used, and to date most successful, therapeutic strategy for the treatment of patients with PAH. The labile nature of PGI2 has meant that such strategies have relied upon the development of stable analogues of PGI2 such as Iloprost, which unfortunately is not orally active and has to be inhaled or given as an I.V. infusion^45^. There have been some clinical trials that have combined PGI2 analogues or ET-1 receptor antagonists with compounds that promote the effect of NO^43^. The role of PGI2 in PAH, the therapeutic potential of PGI2 analogues and receptor agonists in the treatment of the disease and potential novel strategies to maximise drug delivery and effect have recently been reviewed in this Journal^3^.

**Figure 3.**
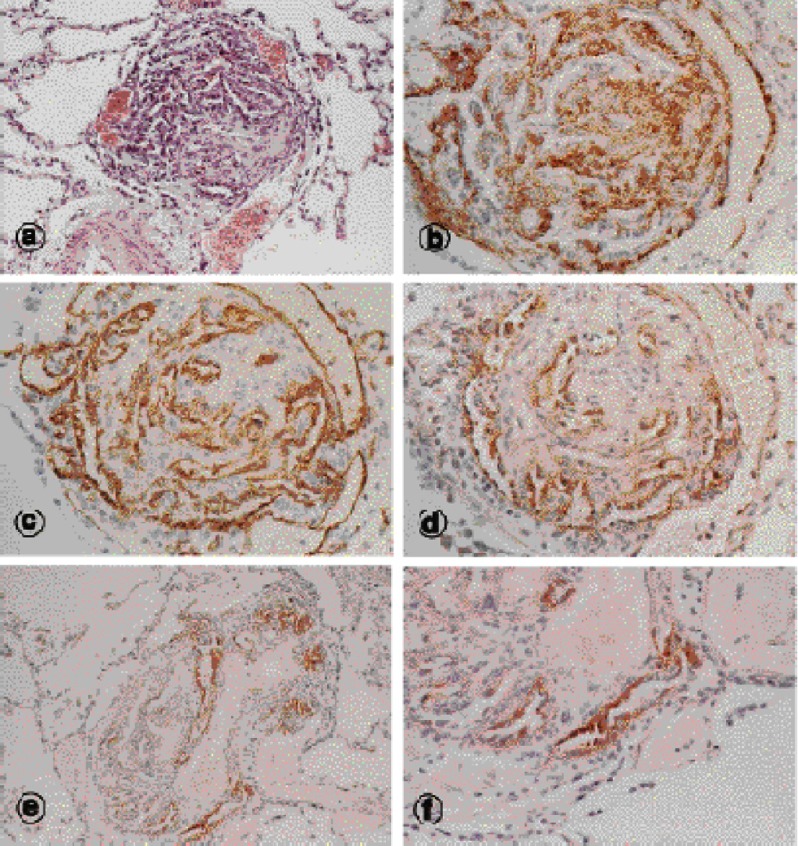
Confocal images of endothelial cells expressing genetically encoded fluorescent probes that specifically respond to NO in (A) the mitochondria (mtC-geNO), (B) the cytosol (G-geNOp) and (C) a merged image of both. Scale bar, 10 mm. (D) Maximal average changes in intensity following the addition of ATP or the NO donor NOC-7 in the mitochondrial (green bar) and the cytosol (grey bar).

In addition to its direct effects on soluble guanylate cyclase, NO has been implicated to play a role in the regulation of mitochondrial function via its ability to interact with components of the electron transport chain^46^. NO has also been shown to activate mitochondrial biogenesis via a cGMP-dependent mechanism^47^. In conditions associated with over-production of NO, such as septic shock, its been shown that low mitochondrial complex I activity is associated with raised nitrite/nitrate concentrations^48^. The irreversible nature of the inhibition of complex I suggest that it is due to the action of reactive nitrogen species, rather than an inhibitory effect of NO. The direct relevance to how NO regulation of mitochondrial function affects the pulmonary vasculature is currently been investigated. In the fetal lung, it has been shown that interactions between endothelial NOS and mitochondria reduce the levels of oxidative stress and facilitates vasodilation at birth^49^. The recent development of fluorescent molecular probes specific for the mitochondria and cytosol, that specifically and directly respond to NO have provided the opportunity to obtain a quantifiable and real-time readout of NO dynamics in different cellular compartments (Figure 3)^50^. This approach may be of use to track the intracellular actions of NO is pulmonary cells.

## Role of nitric oxide in the pathophysiology of PAH

**Figure 4.**
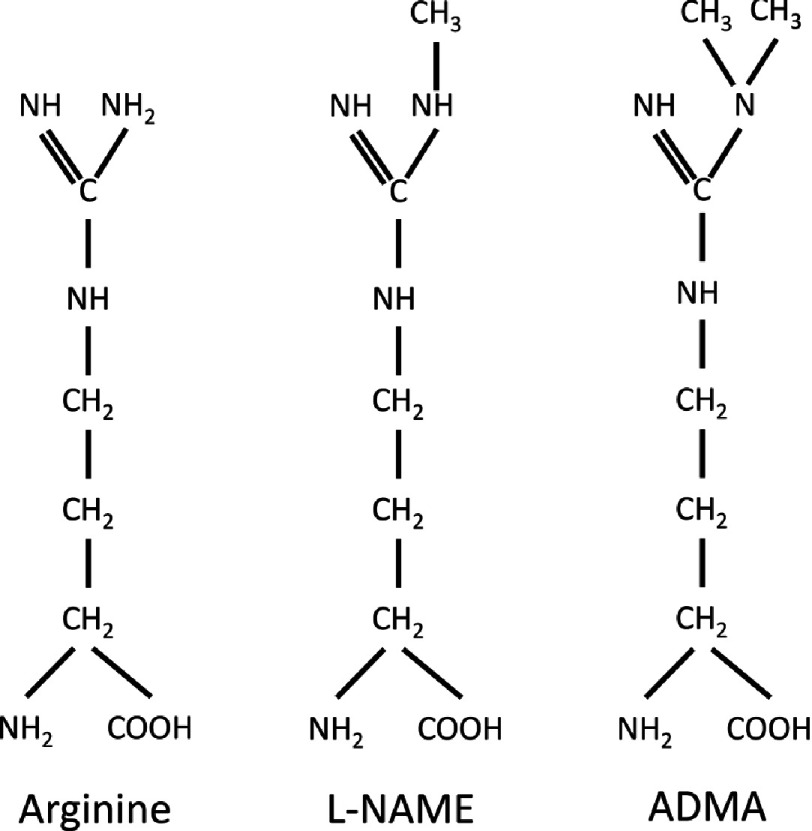
Histological sections of plexiform lesions in pulmonary arteries from patients with PAH (taken from Mason et al.)^53^.

Given the vasodilator effect of NO, it would be easy to speculate that reduced expression or release of NO would be identified in patients with the disease. Indeed there is evidence that pulmonary arteries from patients with PAH due to a variety of reasons have reduced endothelial expression of eNOS^51^. However there are also studies that have shown no change or even increased expression of eNOS in vessels taken from hypertensive lungs^52^. Increased expression of endothelial NOS has been specifically localised to plexiform lesions^53^ (Figure 4). While these studies have been able to show changes in expression of the enzyme responsible for NO synthesis by the endothelium, it remains unclear as to whether this translates to changes in the activity of the enzyme or sensitivity of the vessel wall to the action of NO. Indeed, patients can be classified as responders or non-responders in the ability of inhaled NO to reduce pulmonary vascular resistance (PVR) and mean pulmonary artery pressure (mPAP). Those with a reduction of >30% in PVR or >12% in mPAP were shown to have reduced mortality compared to patients who had responses below these thresholds^54–56^.

Reductions in endothelial NOS have been shown to contribute to impaired mitochondrial biogenesis an ovine model of PAH^57^. Changes in mitochondrial activity in the pulmonary arteries during PAH results in alterations to redox signalling and impaired oxygen sensing. These changes cause activation of transcription factors usually associated with hypoxia, such as hypoxia-induced factor 1a (HIF-1a), which persist even under normoxic conditions^58,59^. HIF-1a can regulate mitochondrial fission, fusion and metabolism of pulmonary artery smooth muscle cells, which contribute to proliferation of smooth muscle cells and remodelling of the vessel wall^60,61^. The regulatory role of NO on mitochondria and the activation of these hypoxia-related pathways remain to be determined.

### Endogenous inhibitors of NOS enzymes

**Figure 5.**
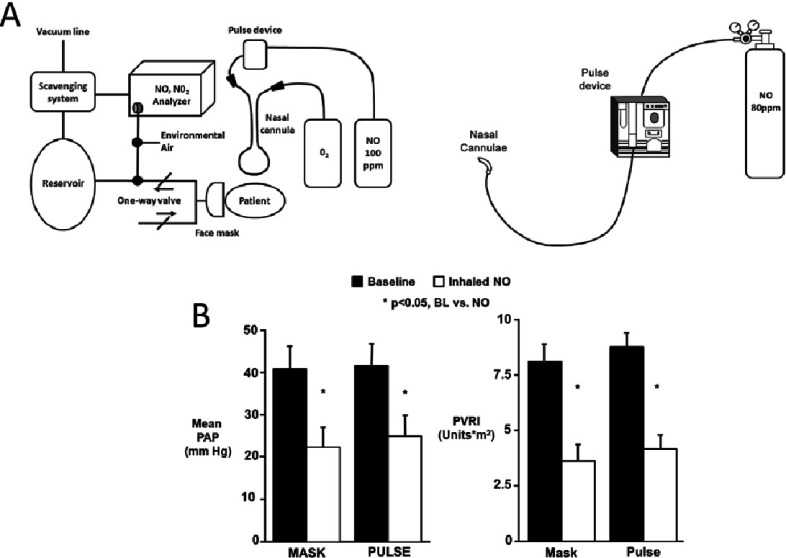
Comparisons of the chemical structure of the nitric oxide synthase substrate arginine with the synthetic inhibitor L-NAME and the endogenous inhibitor ADMA of the enzyme.

Another potential mechanism for impairment of eNOS is the action of circulating inhibitors of NOS enzymes. The first of these inhibitors to be characterised was asymmetric dimethylarginine (ADMA)^56^. ADMA is a methyl derivate of arginine, and is produced by the physiological degradation of methylated proteins (Figure 5). Metabolism of ADMA is mediated by the enzyme dimethylarginine dimethylaminohydrolase (DDAH), forming citrulline and dimethylamine^62^. Loss of DDAH activity leads to accumulation of ADMA and reductions in NO signalling^63^. Functional polymorphisms in the gene for DDAH2 have associated with increased with increased levels of circulating ADMA, suggesting that there may be an association between genetic variations in the expression of DDAH and cardiovascular risk^64,65^. ADMA is made in cells by arginine methylation and is released into the cytosol upon protein degradation^66^. The concentration of ADMA within endothelial cells may be 10–20 fold higher than that seen in the plasma^67^. Plasma levels of ADMA predict cardiovascular events and mortality and have been associated with a wide range of conditions including hyperlipidemia, hypertension, peripheral arterial disease, chronic renal failure, chronic heart failure, diabetes mellitus type II, preeclampsia and pulmonary hypertension^68–71^.

## Nitric oxide as a therapy for PAH

### Inhaled NO gas

**Figure 6.**
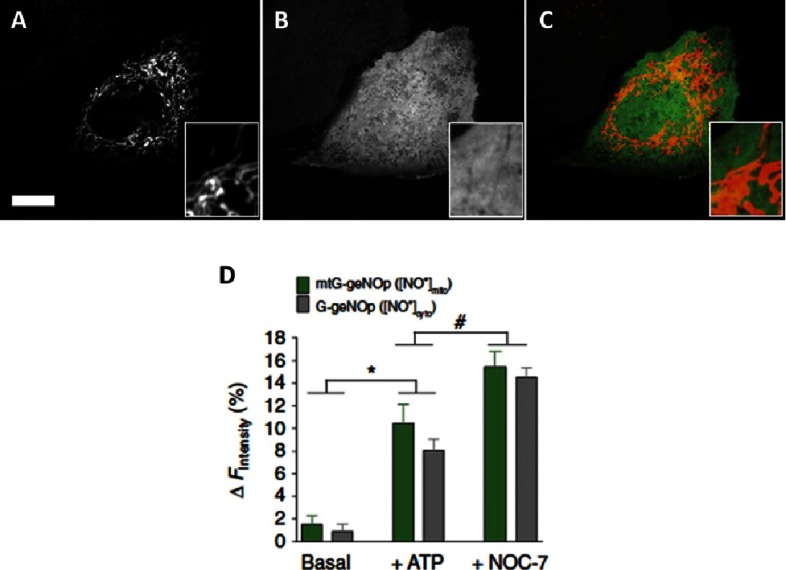
(A) New inhaled NO delivery systems that allow pulsed dosing with the gas and (B) data on the reductions achieved with inhaled NO given by either continuous therapy (MASK) or via a pulsed delivery system (PULSE)^78^.

The vasodilator and anti-proliferative actions of NO make it an attractive tool for pharmacological treatment of PAH. Administration of NO gas by inhalation has been shown to be beneficial to patients with PAH, particularly in paediatric cases^72–74^. However, the usefulness of inhaled NO as a treatment is limited due cost, technical difficulties and the fact that not all patients respond to the therapy. The ability of NO to oxidise haemoglobin to form methaemoglobin, which has been shown to be related the cumulative exposure to the gas, may also limit its effectiveness^75^. Rapid withdrawal of inhaled NO therapy can also have deleterious effects with levels of oxygenation and pulmonary hypertension returning to levels worse than those seen prior to the commencement of therapy^76,77^. However, developments in delivery technology have allowed optimisation of inhaled NO therapy by administering pulsed dosing of the gas (Figure 6A)^78–80^. These studies have shown a reduction in adverse events, no changes in methaemoglobin levels and no reports of syncope^79,81^. The pulsed delivery of NO was shown to be as effective as continuous therapy in reducing mean pulmonary artery pressure and pulmonary vascular resistance (Figure 6B). The use of inhaled NO therapy has recently been shown to be cost effective for the treatment of infants in a trial of NO therapy for chronic lung disease^82^. Despite the cost of the therapy, those patients who received inhaled NO had a shorted hospital stay and ventilation period.

### Phosphodiesterases inhibitors

**Figure 7.**
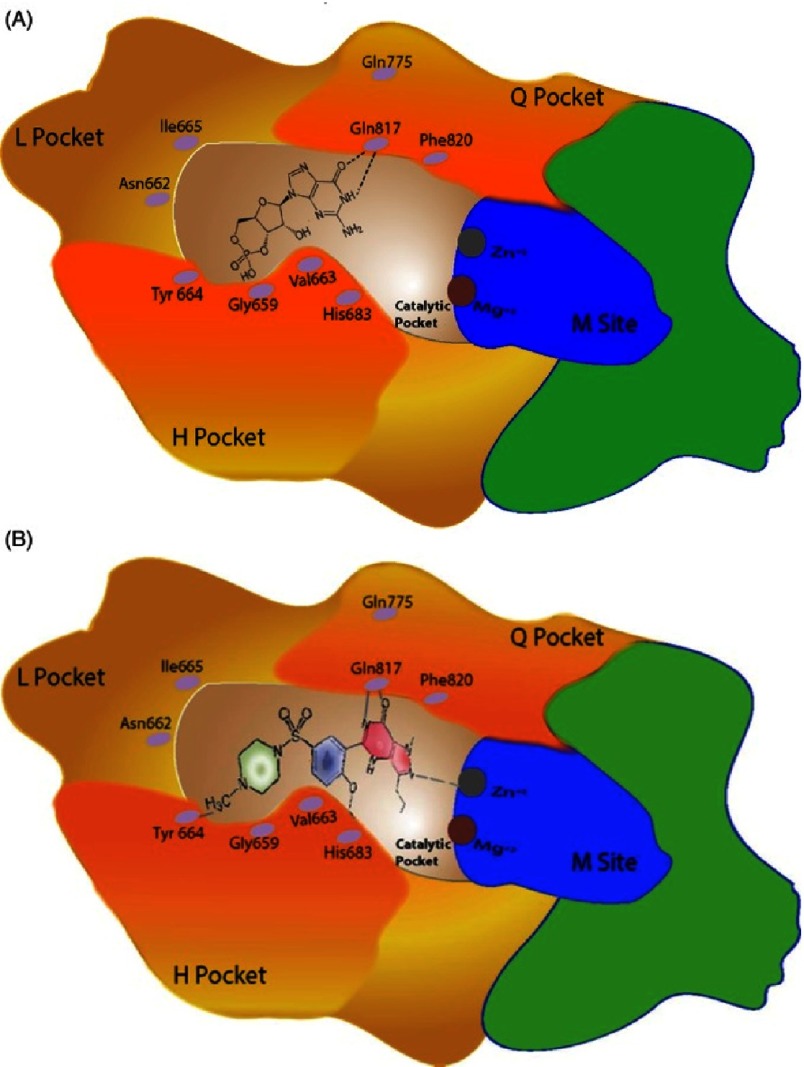
Diagram of catalytic Pocket of PDE showing occupation by (A) cGMP and (B) the PDE5 inhibitor sildenafil^88^.

PDEs are responsible for the degradation of cGMP and thus terminating the effects mediated by NO. There currently 12 different isoforms identifed of these enzymes that differ in their 3-dimensional structures and tissue distribution^83,84^. PDE5 is present in the cardiovascular system; with abundance in the pulmonary vascular smooth muscle and endothelial cells^85,86^. Inhibition of PDE5 has been shown to mediate anti-proliferative effects on pulmonary artery smooth muscle cells^87^. The development of PDE5 inhibitors, such as sildenafil, are effective pharmacological agents for the treatment of patients with PAH due to either ability to bind to the PDE5 enzyme, inhibit the breakdown of cGMP and thereby prolong the effect of endogenous NO (Figure 7)^88^.

The SUPER-1 (Sildenafil Use in Pulmonary Arterial Hypertension) trial showed that sildenafil improves the functional capacity and pulmonary haemodynamics in patients with PAH, with only mild adverse effects^89^. These effects are sustained over a period of 3 years in a follow-up study (SUPER-2)^90^. In addition to sildenafil, tadalafil and vardenafil, two newer PDE5 inhibitors, are currently being assessed for use in patients with PAH. Tadalafil, which is a once a day oral preparation has been shown to improve exercise capacity, pulmonary haemodynamics and time to clinical worsening, effects that last for up to 1 year in the PHIRST-1 trial (Pulmonary Arterial Hypertension and Response to Tadalafil)^91^. Similar results have been reported for vardenafil in the EVALUATION (Efficacy and Safety of Vardenafil in the Treatment of Pulmonary Arterial Hypertension) trial, albeit in a much smaller cohort of patients^92^.

PDE5 is also expressed in the heart and some small studies have suggested that sildenafil is capable of preventing the remodeling of the myocardium seen in heart failure^93,94^. However, in larger clinical trials this effect of sildenafil was not seen^95^. It has recently been suggested that this may be due to the expression of PDE9 in myocytes. While PDE5 is found in contractile filaments, where is degrades cGMP produced by the action of NO on the soluble guanylyl cyclase (sGC) receptor, PDE9 is located near the T-tubular’ invaginations of the plasma membrane^96^. PDE9 in believed to be responsible for the degradation of cGMP produced by atrial natriuretic peptide (ANP) and B-type natriuretic peptide (BNP) hormones acting on the guanylyl cyclase type A (GC-A) receptor that is located the cardiomyocyte plasma membrane.

### cGMP activators

As an alternative to inhibition of the breakdown of cGMP, a new class of drugs are now being developed that directly activates soluble guanylate cyclase, thereby mimicking the effects of NO by increasing levels of cGMP. One such drug is riociguat, which has been assessed for efficacy in the treatment of PAH^97^. Results of the PATENT-1 (The Pulmonary Arterial Hypertension Soluble Guanylate Cyclase-Stimulator Trial) trial showed an improvement in exercise capacity, pulmonary haemodynamics, N-terminal pro-brain natriuretic peptide levels and time to clinical worsening. While adverse effects were similar to PDE5 inhibitors there was a greater incidence of systemic hypotension and haemoptysis.

### Combination therapy

The mainstay of treatment for PAH is still the use of prostacyclin mimetic compounds and more recently endothelin-receptor antagonists and PDE-5 inhibitors. There are potential advantages in using combinations of these drugs to enhance the clinical benefits that can be obtained when the drugs are used in isolation. The use of strategies that combine treatment with PDE-5 inhibitors with PGI2 analogues and/or ET receptor antagonists have recently been comprehensively reviewed^43^. The results of some of the trials of combination therapy do show some promise, however some results are contradictory. For example the COMPASS-2 (Combination of Bosentan and Sildenafil versus Sildenafil Monotherapy on Pulmonary Arterial Hypertension) trial was able to show an improvement in the 6-minute walk test after 16 weeks of treatment but failed to delay the time to the first morbidity/mortality event^98^. However, meta-analysis of data from studies that involve combination therapy do indicate that this approach is effective in the treatment of PAH^9^.

### Future therapies

**Figure 8.**
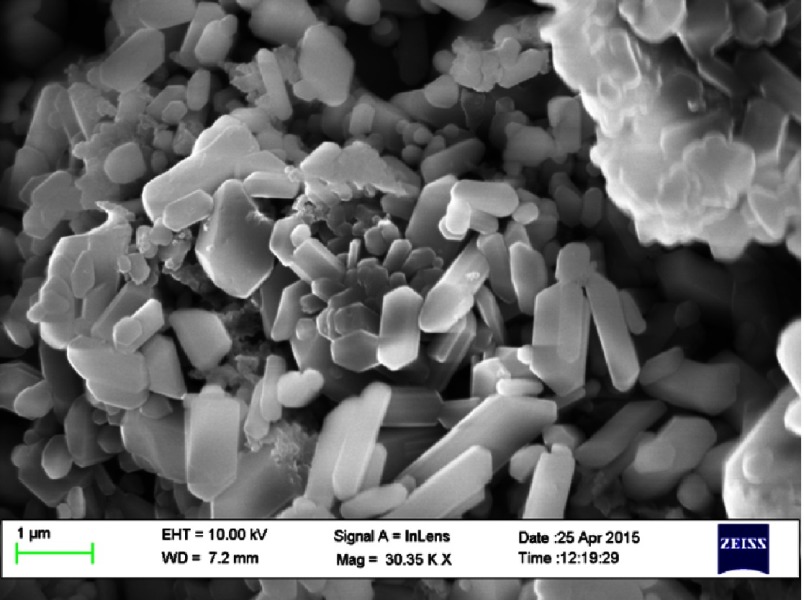
Scanning electron micrograph on NO-containing nano-particles.

The current pharmacological strategies to augment the supply or duration of the action of NO have a number of limitations mainly relayed to the routes of administration, duration of action and systemic effects. Novel nanoparticles are being developed for the treatment of a number of different conditions (Figure 8)^99,100^. Of particular interest are fibres that are capable of releasing NO and that are suitable for delivery via inhalation to deliver NO directly to the pulmonary vasculature^101,102^. These nanofibres have recently been shown to be able to continuously release NO over an 8-hour period and relax pulmonary vessel of rats with pulmonary hypertension *in vitro*^103^. These initial observations suggest that nanofibres capable of releasing NO may be of use in the future treatment of patients with PAH.

## Conclusions

NO plays a significant role in the pulmonary circulation. Changes to its synthesis, release or signalling contribute to the pathogenesis of PAH. While targeting NO in has resulted in advances the treatment of the disease, the quality of life and the prognosis for patients with PAH remains poor. There remains a substantial amount of work to be done in order to optimise strategies that target to NO system or utilise the delivery of NO as a pharmacological agent.
